# A matter of faith…: Dr. D.K. Karanjavala

**DOI:** 10.4103/0970-1591.40603

**Published:** 2008

**Authors:** Anita Patel

**Affiliations:** Consultant Urologist, Endoskopik Klinik & Hospital, Mumbai, India

**Figure:**
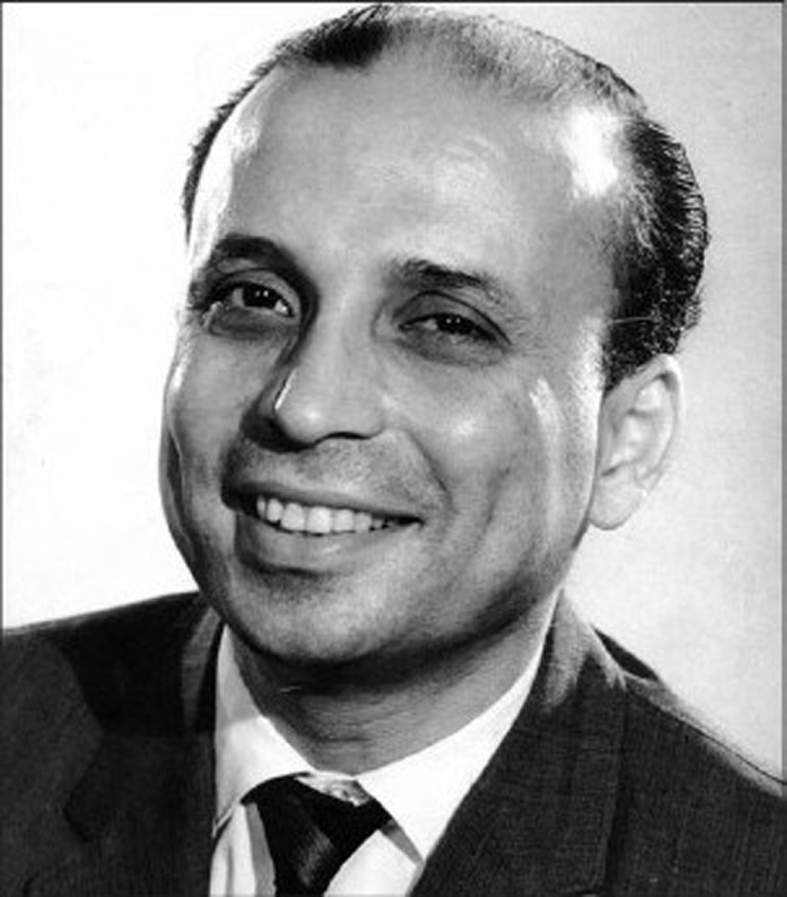
Dr. D.K. Karanjavala

“If I were to choose a career today, it would still be urology, provided it is practiced the way we did!” asserts the octogenarian, Dr D.K. Karanjavala.

Recipient of three prestigious awards including Padmashri in 1983 [Government of India], Dhanwantari in 1986 [Government of Maharashtra], and St. Paul's Gold medal from British Association of Urological surgeons (BAUS) in mid 80s; the man at the ripe age of 88 is a true legend in Indian Urology.

Born in 1920, in Mumbai, he spent his early childhood at a boarding school in Panchgani, where strong values of caring and respect for humanity were inculcated in him. His inherent fondness for science drew him to medicine as a specialty. The undergraduate days saw him winning many laurels and he graduated in 1944 from KEM Hospital in Mumbai and later acquired his MS in General Surgery from the same hospital in 1948. As was customary in those days, going to UK for obtaining an FRCS in surgery was the next logical step.

The following 7 years spent in various hospitals, mainly in and around London, gave him a wide exposure to various surgical subspecialties. However, he attributes his interest in urology to two people, mainly Mr. Terrence Millins (a legendary figure of in world urology, who standardized the technique of “Millin's Retropubic Prostatectomy) and Mr. A.W. Badenoch (a doyen in world urology who established the techniques of open surgery for prostatic and urethral obstruction).

On his return to India in 1955, despite his Indian and British qualification, there was some initial struggle to get recognition. By the end of 1958, he decided to restrict his practice to urology, for which he was ridiculed by many! He formally joined KEM Hospital (Parel, Mumbai), and Bombay Hospital (Marine Lines, Mumbai) in 1958 as a consultant urologist.

He frequently visited Kashmir during his early years as urologist and helped the Government of Kashmir to establish a department of urology at Sher-e-Kashmir Institute at Srinagar. Later he was also appointed as a visiting consultant urologist at Gujamal Modi Hospital and Research Centre for Medical Sciences, Delhi.

Interestingly, his early years in practice saw him treating many veterinary ‘patients’ also, with abdominal and urological diseases!

He was (and still is) a very keen academician and ensured that he attended as many clinical meetings as possible. In the days where fancy imaging techniques (sonography and computerized tomography, considered basic today) were not invented, clinicians depended heavily on history and examination findings; and it is these skills that established his name in the field of urology nationwide. His list of patients included the who's who in the field of politics, arts, and science in India. Practicing urology before the era of subspecialization meant one had to be a jack of all! Apart from surgical treatment of prostate disease, Dr. Karanjavala developed special interest in pediatric urology and reported some of his findings in children with meningomyelocoele, in *Indian Journal of childhood* in 1958. He also presented his experience in management of pediatric hydrocolpos and mucocolpos in the BAUS in 1969.

He took keen interest in investigation and surgical treatment of chyluria. In fact this helped him attain international acclaim with his papers on the same being published first in *Indian Journal of Surgery* in 1966 and in the *British Journal of Urology* (1979), and *Annals of Royal College of Surgeons* (1970).[1–3]

He was selected as the national delegate (India) at the Society Internationale D'urologie (SIU) in 1958 and held that position till 1982. He was elected as the President of Urology Society of India (USI) in 1981. In mid-seventies, he was also elected to the editorial board of the *British Journal of Urology*.

A sudden myocardial infarct in 1980 enforced a period of rest on him, eventually necessitating a coronary bypass a few months later. He slowed down considerably thereafter and formally retired from Bombay Hospital at the ripe age of 73 (1993).

Besides urology, Dr. Karanjavala is very fond of good food and travel. His urological prominence and academic pursuits have taken him to every corner of the world, thus helping him build a large international family of some very prominent people. He is blessed with three children (and several grandchildren); all happily married and settled in different parts of the world. His lovely wife, Khorshed, unfortunately passed away in 2005 after a brief illness.

Today sitting back in the twilight of his life, he believes that the success of his practice has been largely due to the mutual faith between him and his patients. He cautions the young urologists of today, not to become toys at the hands of technology. After all it is the human aspect of treatment, which makes medical practice so unique and it is up to us to maintain that dignity forever.
